# In Vivo Magnetic Resonance Imaging and Microwave Thermotherapy of Cancer Using Novel Chitosan Microcapsules

**DOI:** 10.1186/s11671-016-1536-0

**Published:** 2016-07-15

**Authors:** Shunsong Tang, Qijun Du, Tianlong Liu, Longfei Tan, Meng Niu, Long Gao, Zhongbing Huang, Changhui Fu, Tengchuang Ma, Xianwei Meng, Haibo Shao

**Affiliations:** Department of Radiology, the First Hospital of China Medical University, No. 155 Nanjing North Road, Shenyang, 110001 People’s Republic of China; Laboratory of Controllable Preparation and Application of Nanomaterials, Center for Micro/Nanomaterials and Technology and Key Laboratory of Photochemical Conversion and Optoelectronic Materials, Technical Institute of Physics and Chemistry, Chinese Academy of Sciences, Beijing, 100190 China; College of Materials Science and Engineering, Sichuan University, Chengdu, 610065 China

**Keywords:** Microwave, Thermotherapy, Chitosan, Microcapsules, MR imaging, Ionic liquids

## Abstract

Herein, we develop a novel integrated strategy for the preparation of theranostic chitosan microcapsules by encapsulating ion liquids (ILs) and Fe_3_O_4_ nanoparticles. The as-prepared chitosan/Fe_3_O_4_@IL microcapsules exhibit not only significant heating efficacy in vitro under microwave (MW) irradiation but also obvious enhancement of T_2_-weighted magnetic resonance (MR) imaging, besides the excellent biocompatibility in physiological environments. The chitosan/Fe_3_O_4_@IL microcapsules show ideal temperature rise and therapeutic efficiency when applied to microwave thermal therapy in vivo. Complete tumor elimination is realizing after MW irradiation at an ultralow power density (1.8 W/cm^2^), while neither the MW group nor the chitosan microcapsule group has significant influence on the tumor development. The applicability of the chitosan/Fe_3_O_4_@IL microcapsules as an efficient contrast agent for MR imaging is proved in vivo. Moreover, the result of in vivo systematic toxicity shows that chitosan/Fe_3_O_4_@IL microcapsules have no acute fatal toxicity. Our study presents an interesting type of multifunctional platform developed by chitosan microcapsule promising for imaging-guided MW thermotherapy.

**Electronic supplementary material:**

The online version of this article (doi:10.1186/s11671-016-1536-0) contains supplementary material, which is available to authorized users.

## Background

In recent years, microwave (MW) thermotherapy received considerable attention due to many advantages including the ability to achieve effective ablation of large tumors and less heat sink effects, when compared with other cancer therapies such as radiotherapy, chemotherapy, and surgery [1–6]. For the clinical application of MW thermotherapy, the main challenge is achieving a balance between complete ablation of tumor tissue and minimum damage to surrounding vital organs [7–11]. Nanomaterials have emerged with the unique characteristics of localization of the MW irradiation, which results in improving the efficacy of MW hyperthermia and eliminating the limitations. Carbon nanotubes and ferrite compounds have been utilized as MW agents for MW thermotherapy [12–16], whose heating efficacy was too low to use in vivo. Microwave thermal therapy uses dielectric hysteresis to produce heat. Heating of the tissue is based on agitation of water molecules or ions under an electromagnetic field of microwave frequency, rather than relying upon current flow and resistive heating [17]. Two main mechanisms are identified that trigger heating by microwave irradiation: dipolar polarization mechanism and ionic conduction mechanism [18–20]. The ionic conduction process represents a much higher heat-generating efficacy than the dipolar polarization. Because of their ionic character and high polarizability, room-temperature ionic liquids (ILs) are extremely susceptible to microwave irradiation [21–25]. As excellent absorbers, ILs show highly efficient microwave heating, which provide superior benefits for the application of tumor MW thermotherapy. In order to deliver the liquid-phase absorbers to the tumor site, encapsulation of the ILs with biocompatible microcapsules is one of the most promising resolutions. The microcapsules have a compact shell structure, which can confine the oscillation of IL leading to more serious friction and collision of molecules and ions than the free IL. Thus efficient, rapid, and selective heating occurs in the microcapsules.

Chitosan-based materials have sparked considerable interest due to their good biocompatibility, biodegradability, and low production costs, all of which result in their potential use in various biological and clinical applications, including slimming, wound dressing, and tissue engineering, especially those involving drug delivery [26–29]. Chitosan is composed of amido and hydroxyl groups, which can be combined with polymer and metal. Bhise et al. have designed sustained release systems for the anionic drug naproxen using chitosan as drug carrier matrix [30]. Sun et al. have designed enoxaparin/chitosan nanoparticulate delivery systems, providing very stable complexes that led to a significantly improved drug uptake [31]. Guo et al. have developed chitosan-coated hollow CuS nanoparticles that assemble the cytosine-guanine oligodeoxy nucleotides for photothermal therapy in a mouse breast cancer model [32]. As compared to chitosan particles with single modality, dual modalities with both the therapeutic and imaging function offer great potential to satisfy the increasing requirements in advanced tumor theranostic platform. In the platform, the imaging modality can be used as contrast agents to light up tumors and the therapeutic modality can be used to destroy tumors. However, success on preparation of chitosan microcapsules with payload of MRI contrast agents and MW absorbers for imaging-guided MW thermotherapy, to best of our knowledge, has not yet been reported.

In this work, we have developed a facile strategy to construct chitosan-based microcapsules as MW susceptible agent for tumor thermotherapy and contrast agents for enhanced tumor MR imaging. The chitosan/Fe_3_O_4_@IL microcapsules showed high MW conversion efficiency in vitro MW heating experiment. In vitro and in vivo toxicity results uncovered that these microcapsules exhibited good biocompatibility. For use in a mice model, the as-prepared microcapsules showed dramatically enhanced MW thermotherapy outcomes in vivo. It is the first report that using chitosan/Fe_3_O_4_@IL microcapsules to kill the H22 tumor cells in mice with 100 % of tumor elimination. In vivo imaging results demonstrated that these chitosan/Fe_3_O_4_@ILmicrocapsules can achieve MR imaging-guided MW thermotherapy. Our study highlights the great potential of chitosan-based microcapsules for cancer theranostic applications.

## Methods

### Materials

Ethylene glycol, ferric chloride hexahydrate (FeCl_3_·6H_2_O), sodium acetate (NaAc), sodium citrate, acetate, glutaraldehyde, and chitosan (deacetylation degree = 80–95 %) were purchased from Sinopharm Chemical Reagent Beijing Co., Ltd. Soybean oil was obtained from Beijing Guchuan Oil Co. Span 80 was purchased from Institute of Tianjin Jinke Fine Chemicals. 1-Butyl-3-methylimidazolium tetrafluoroborate (IL) was purchased from Shanghai Chengjie Chemical Co., Ltd. Mercaptopropionic acid (MPA) was obtained purchased from Alfa Aesar. Hematoxylin and eosin (H&E stain) were obtained from Beijing Solarbio Science and Technology (China). All chemicals were used directly in our work without further purification.

### Preparation of Fe_3_O_4_ NPs

The Fe_3_O_4_ NPs were prepared by a gradient solvothermal method. In a typical methods, 0.5 g FeCl_3_·6H_2_O was dissolved in 5 mL ethylene glycol under stirring. Then, 0.15 g sodium citrate and 0.9 g NaAc were completely dissolved in 10 mL ethylene glycol. And the above solutions were mixed together under stirring and the pH value was adjusted to 7. The solutions were translated into a 50-mL Teflon lined stainless steel autoclave and heated at 200 °C for 4 h. The black product was magnetically collected and rinsed for four times with deionized water and absolute ethanol, respectively, and then dried at 50 °C for 24 h.

### Preparation of Chitosan/Fe_3_O_4_@IL Microcapsules

**Fig. 1:**
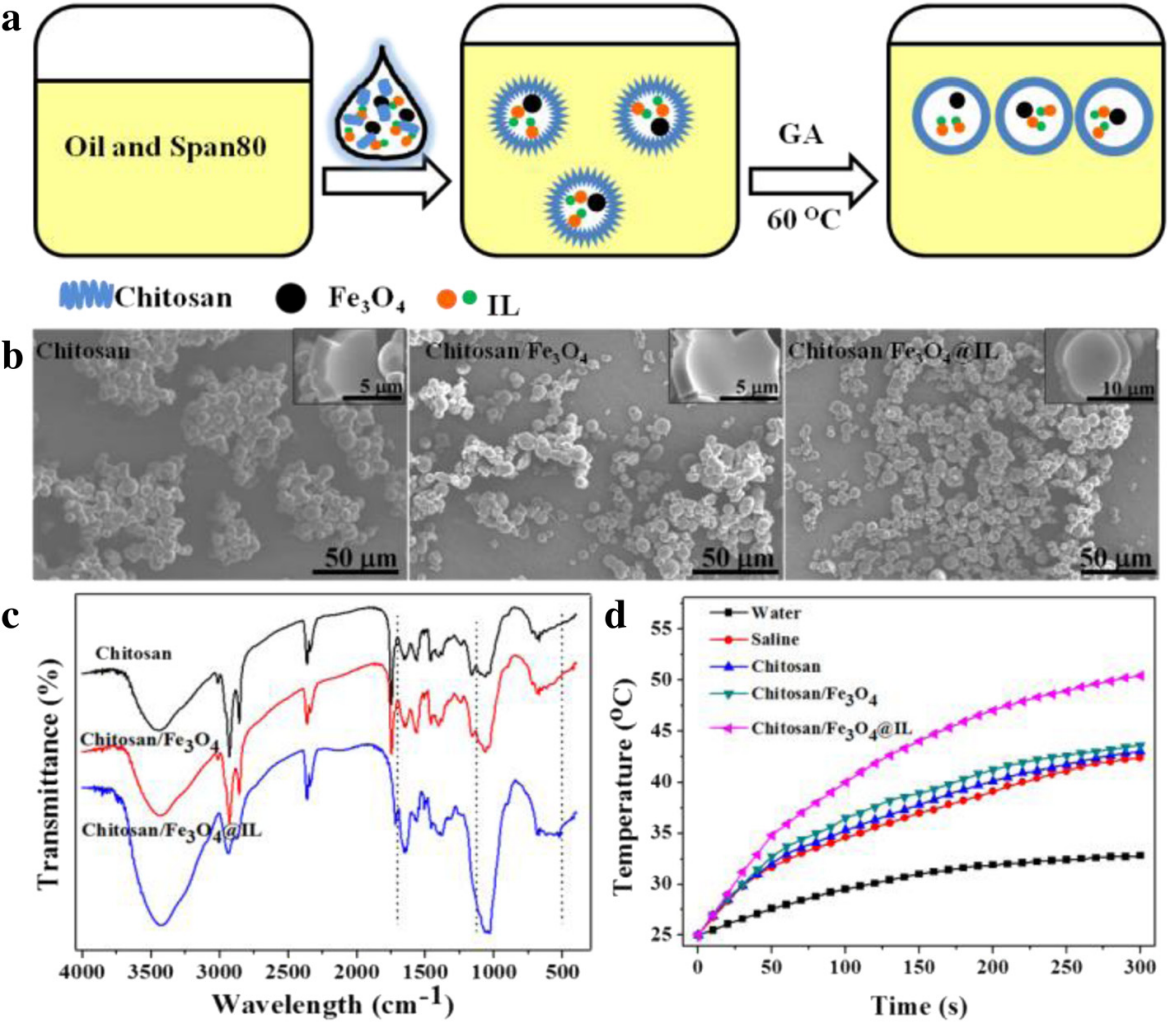
Chitosan synthesis and characterization. **a** A schematic illustration of microcapsules synthesis. **b** SEM images of chitosan, chitosan/Fe_3_O_4_, and chitosan/Fe_3_O_4_@IL microcapsules. **c** FT-IR spectra of chitosan, chitosan/Fe_3_O_4_, and chitosan/Fe_3_O_4_@ILmicrocapsules. **d** Heating curves of water, saline, and microcapsules under MW irradiation at a power density of 1.8 W/cm^2^ for 5 min

Chitosan was completely dissolved in deionized water with 3 % acetate, 0.02 g Fe_3_O_4_ NPs, and 8 % ILs at a concentration of 0.045 g/mL and marked as water phase. Fifty-milliliter soybean oil and 1.2 mL Span 80 were mixed well and marked as oil phase. Ten-milliliter water phase was slowly added into oil phase under stirring at 1200 r/min for 10 min to form water/oil emulsion (Fig. 1a). Then, 1 mL 50 % glutaraldehyde was added into the emulsion and heated at 60 °C for 60 min. Finally, the microcapsules was collected by washing for four times with deionized water and absolute ethanol, respectively, followed by drying under 45 °C for 24 h.

### Characterization

The morphology of as-prepared microcapsules was characterized by scanning electron microscopy (SEM, S-4300, Hitachi). Energy dispersive spectroscopy (EDS) was also recorded by Hitachi S-4800 SEM. The surface functional groups of the microcapsules were employed by Fourier transform infrared spectrometry (FT-IR, Varian, Model 3100 Excalibur). Thermogravimetric analyses were performed on a thermal analyzer (TG, ICES-001, Canadian) in the range 25–800 °C with a heating rate of 10 °C/min in nitrogen atmosphere. Canon DS126231 digital camera was recorded to take the tumor photographs. The UV/vis absorption spectra were measured by a spectrophotometer (JASCO V-570) at room temperature. Magnetic measurements were investigated by a magnetometer (Model PPMS-9).

### In Vitro MW Heating Experiment

In the MW heating experiment, the as-prepared microcapsules were diluted to 1 mL by saline solution at a concentration of 50 mg/mL. Then, the solution was added into a 12-well plate with a thin bottom and exposed to MW irradiation (1.8 W, 5 min, Beijing Muheyu Electronics Co., LTD). The temperature of the solution was detected with a fiber thermometers (Beijing Dongfangruizhe Technology Co., LTD).

### In Vitro Cytotoxicity

Fresh blood was collected from rabbit’s heart in a 10 mL centrifuge tube, containing EDTA as the anticoagulant (anticoagulant to blood ratio, 1:9, *v*/*v*). The whole blood was centrifuged and washed with PBS for three times and rediluted in PBS, to give a 2 % erythrocyte suspension. 0.5 mL microcapsules were added into glass tubes, followed by the addition of 0.5 mL erythrocyte suspension and kept at room temperature for 3 h. Subsequently, all the samples were centrifuged at 10,000 rpm for 3 min. The hemoglobin released from the erythrocytes in the supernatant was determined at 570 nm by an ultraviolet spectrophotometer. Distilled water and PBS solution were used as a positive control (100 % lysis) and negative control (0 % lysis), respectively. The content of hemolysis rate (%) was determined as following formula: Hemolysis rate (%) = (tested sample − negative control)/(positive control − negative control) × 100. Triplicate samples were repeated and the values were averaged.

The viability of cells were evaluated by 3-(4,5-dimethylthiazol-2-yl)-2,5-diphenylte-trazolium bromide (MTT) assays. Briefly, HepG2 cells were plated out at a concentration of 4 × 10^4^ cells/mL in 96-well plate and incubated for 24 h at 37 °C with 5 % CO_2_. Then, different concentrations of microcapsules were added and incubated for 24 h. The control group was only incubated with HepG2 cells. MTT-phosphate saline-buffered solution (20 μL) was then added and incubated for another 4 h. Finally, the medium were removed and 150 mL dimethyl sulfoxide (DMSO) was added into each well. The absorbance of the suspension was recorded by a scanning multiwall spectrometer (Multiskan MK3 Thermo). The cell viability (%) = (absorbance of experimental)/(absorbance of control groups) × 100. All of the experiments were repeated five times for each group.

### MW Heating Therapy

Experimentation with animals was governed by the Regulations of Experimental Animals of Beijing Authority and approved by the Animal Ethics Committee of the Peking University.

All ICR mice were obtained from Vital River Laboratory Animal Technology Co. Ltd., Beijing, and used under the guidelines of the Institutional Animal Care. An infrared thermal mapping apparatus (FLIR SC620) with MW apparatus (Beijing Muheyu Electronics Co., LTD) were used to irradiate tumors in our experiment. To develop H22 tumors, 10^7^ H22 cells (0.1 mL) were injected in the right axillary region of each mice. When the size of the tumor reached to the 300 mm^3^, microcapsules were intratumorally injected at the concentration of 200 mg/kg. After the injection of microcapsules for 1 hour, the tumor of each mouse was then irradiated by the MW at the power density of 1.8 W/cm^2^ for 5 min. There were four groups of mice (control, MW, chitosan/Fe_3_O_4_@IL + MW, chitosan/Fe_3_O_4_@IL) with five mice for each group. The tumor sizes were measured by a vernier caliper every 3 days and calculated as the volume (V) = (length) × (width)^2^/2. After 17 days of therapy, mice were sacrificed and the tumor tissues in each group were weighed.

### MR Imaging

T_2_-weighted MR imaging was obtained by using a 3.0 T MR imaging instrument at room temperature (Signa HDx; General Electric Medical Systems, USA). Dilutions of microcapsules in water with different concentrations as a contrast agent were placed in 4.0 mL eppendorf tubes for T_2_-weight MR imaging. Relativity values of *R*_2_ were calculated through the curve fitting of 1/T_2_ relaxation time versus the microcapsules concentration. For in vivo MR imaging, 10-mg microcapsules were dispersed in 0.2 mL saline solution, then chitosan/Fe_3_O_4_@IL microcapsules (200 mg/kg) were intratumoral injected into the H22 tumor-bearing mice and then taken for the T_2_-weighted MR imaging tests.

### In Vivo Systematic Toxicity

A total of 20 healthy ICR mice were randomly divided into three groups and injected subcutaneously in the axillary region with 2000 mg/kg, 200 mg/kg, and without treatment. Body weight was measured every 3 days by electronic scale. After 17 days, mice were sacrificed for performing a full necropsy. Organs (including liver, spleen, lung, and kidney) were harvested, fixed in 10 % formalin, embedded in paraffin, sectioned, stained with hematoxylin and eosin (H&E) for histological examination using standard techniques, and observed by a optical fluorescence microscope (Nikon Eclipse Ti-S, CCD: Ri1).

## Results and Discussion

### Synthesis and Characterization of Microcapsules

The experimental route to fabricate chitosan/Fe_3_O_4_@IL is presented in Fig. 1a. Starting from Fe_3_O_4_ NPs prepared following a gradient solvothermal method, we synthesized chitosan/Fe_3_O_4_@IL microcapsules by a single coacervation route. The chitosan was cross-linked by GA to form microcapsules. The morphological characterizations of the chitosan, chitosan/Fe_3_O_4_, and chitosan/Fe_3_O_4_@IL microcapsules were evaluated by SEM (Fig. 1b). The microcapsules had similar metric, an almost spherical shape. Shown in Additional file 1: Figure S1 are the size distributions of microcapsules, obtained by more than 200 samples in Fig. 1b. The results indicated that the mean diameters of chitosan, chitosan/Fe_3_O_4_, and chitosan/Fe_3_O_4_@IL microcapsules were 4.95, 5.19, and 6.17 μm, respectively.

FT-IR analysis was carried out to confirm the possible groups of chitosan microcapsules. As shown in Fig. 1c, the characteristic absorption peaks at 1634, 1063, and 523 cm^−1^ were only observed in the chitosan/Fe_3_O_4_@IL microcapsules, which are assigned to C–C stretching vibrations of the imidazolium ring, asymmetric stretching vibrations of BF_4_^−^ and out-plane C–H bending vibrations of imidazolium ring, respectively [33, 34]. These distinct peaks suggest the presence of IL. The elemental in microcapsules was carried out by EDS. Additional file 1: Figure S2 shows the EDS spectra of chitosan microcapsules, and only in chitosan/Fe_3_O_4_@IL microcapsules shows the existence of F element. Moreover, as presented in Additional file 1: Figure S3, after the final thermal decomposition, the percentages of weight of the chitosan, chitosan/Fe_3_O_4_, and chitosan/Fe_3_O_4_@IL microcapsules were 16.49, 21.54, and 23.79 %, respectively. The higher residual of content of the chitosan/Fe_3_O_4_ and chitosan/Fe_3_O_4_@IL is certainly attributable to the Fe_3_O_4_ and Fe_3_O_4_@IL in the microcapsules, respectively.

An important feature of IL was the MW-induced thermal effect, which could be used for ablation of tumors. The temperature elevation of microcapsules was investigated under MW irradiation (1.8 W/cm^−2^) for 5 min. Figure 1d shows the temperature of the aqueous dispersion of microcapsules as a function of irradiation time. A rapid temperature increase of the chitosan/Fe_3_O_4_@IL microcapsules when exposed to MW irradiation was observed up to a high level (ΔT = 25.4 °C). However, the water, saline, chitosan, and chitosan/Fe_3_O_4_groups showed much less temperature change (ΔT = 7.8 °C, ΔT = 17.4 °C, and ΔT = 17.5 °C, respectively), suggesting that either saline or chitosan/Fe_3_O_4_ microcapsules had less effect on the temperature elevation. MW could directly excite ILs in chitosan microcapsules, which led to significantly heating [35–39]. The temperature elevation suggested that chitosan/Fe_3_O_4_@IL microcapsules could act as an efficient MW susceptible agent for tumor ablation.

### In Vitro Cell Experiments

**Fig. 2:**
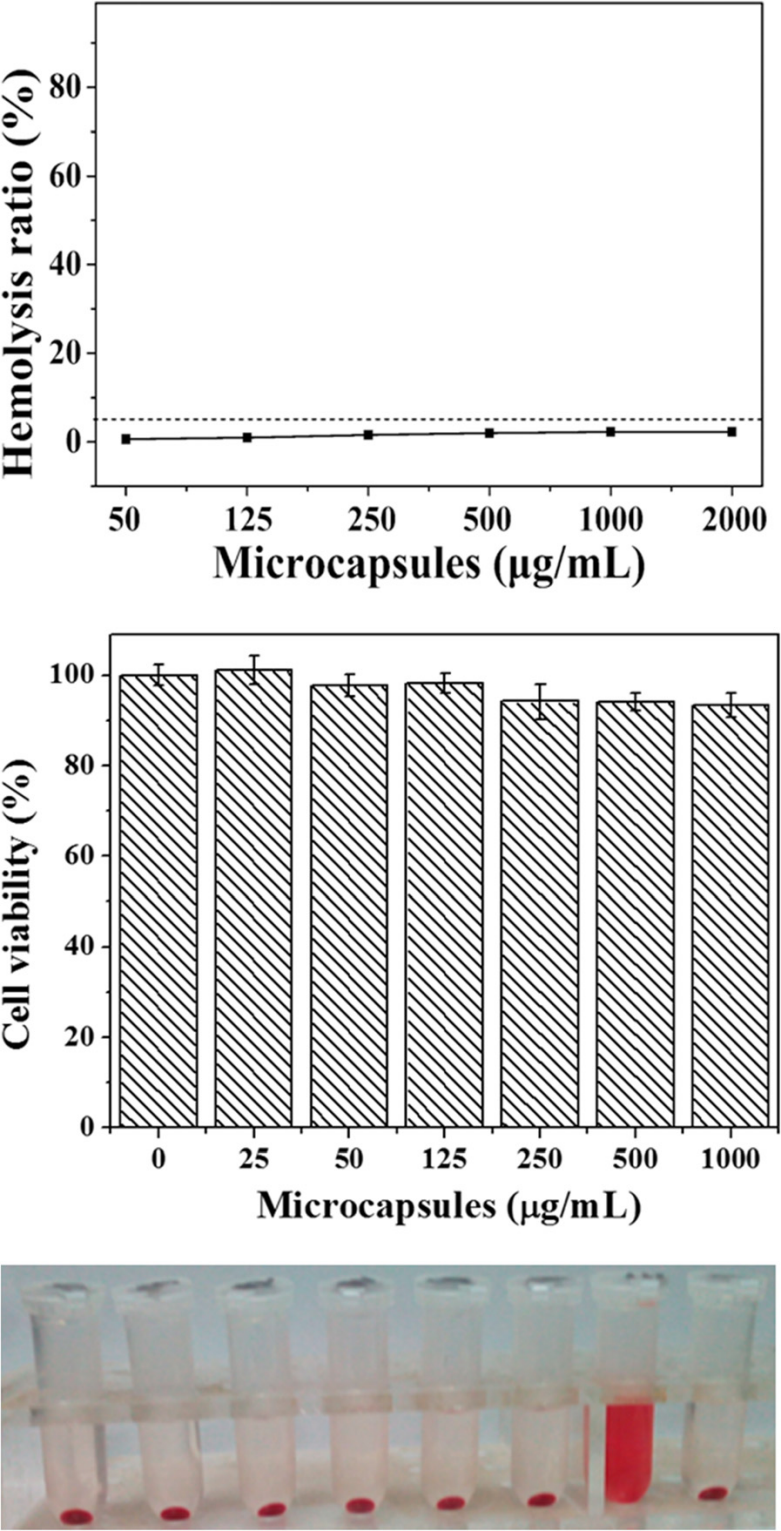
In vitro cytotoxicity. **a** Hemolyticrate of red blood cells incubated with chitosan/Fe_3_O_4_@IL microcapsules at various concentrations for 3 h, using PBS (−) and deionized water (+) as negative and positive controls, respectively. *Inset*: the photo images for direct observation of hemolysis for microcapsules. **b** Viabilities of HepG2 cells incubated with chitosan/Fe_3_O_4_@IL microcapsules with different concentrations for 24 h

Before using the synthesized chitosan/Fe_3_O_4_@IL microcapsules for biomedical application, we firstly evaluated microcapsules in vitro toxicity to cells. Herein, hemolytic behavior of red blood cells was carried out to evaluate its biocompatibility, where deionized water and phosphate-buffered saline were defined as positive and negative controls, respectively. As presented in Fig. 2a, the hemolysis rates of all the samples were below 5 %. The photographs of hemolysis results show that almost no hemolysis of red blood cells can be detected, suggesting that their good blood compatibility (inset of Fig. 2a). Moreover, viabilities of HepG 2 cells were measured after incubating with chitosan/Fe_3_O_4_@IL at tested concentration. According to the MTT assay, approximately >93 % of the cells remained viable after being exposed to the microcapsules for 24 h. Therefore, the hemolytic activity and low cytotoxicity demonstrate that chitosan/Fe_3_O_4_@IL microcapsules exhibits excellent biocompatibility and thus can serve as a promising platform for cancer treatment.

### In Vivo Cancer MW Therapy

**Fig. 3:**
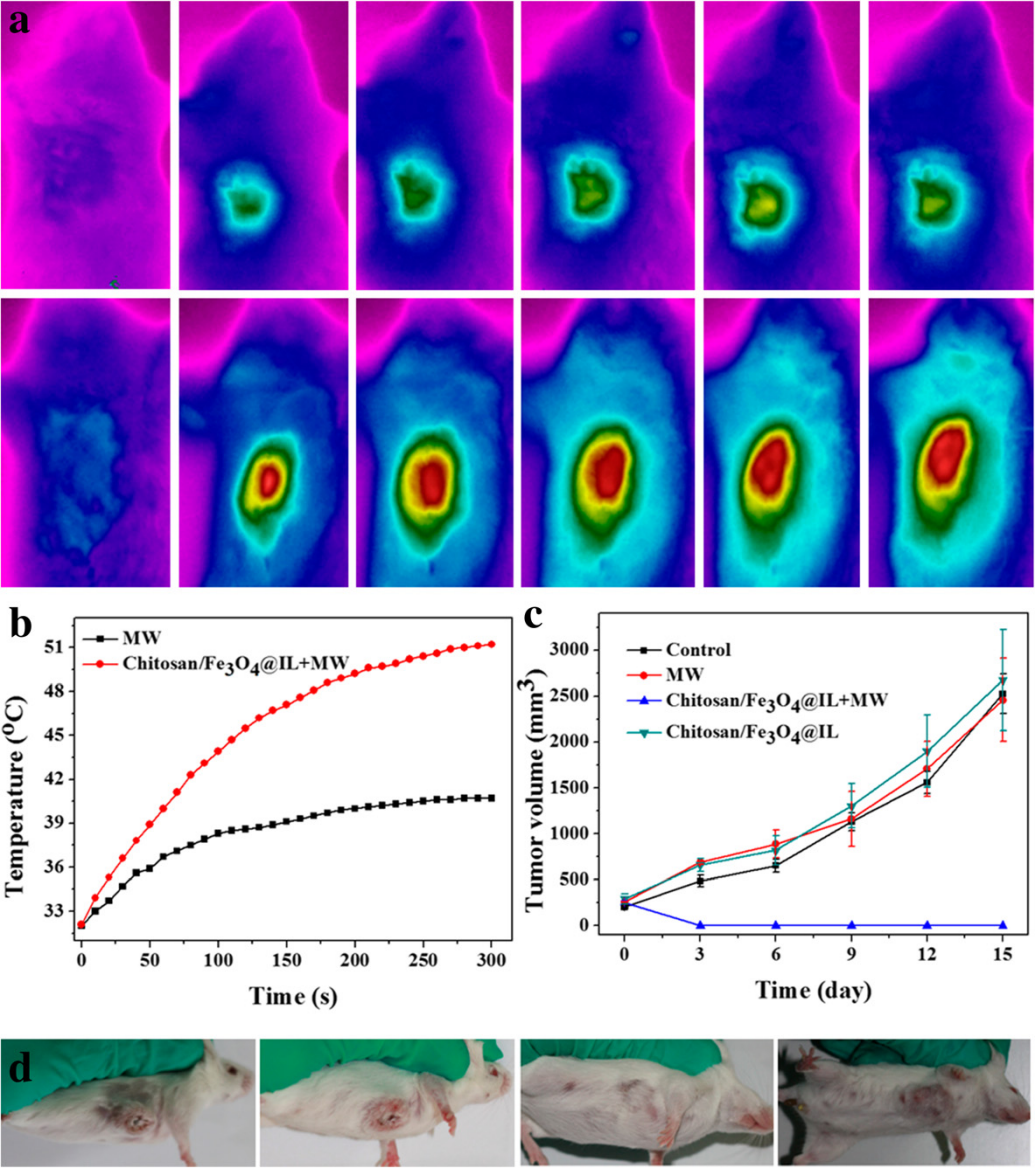
In vivo MW therapy. **a** IR thermal imaging of tumor-bearing mice injected with none and chitosan/Fe_3_O_4_@IL microcapsules under MW irradiation (1.8 W/cm^2^, 5 min). **b** The temperature changes on tumor of mice under different treatment in **a. c** Tumor growth curves of different groups of tumor after various treatment. **d** Representative photos of mice bearing H22 tumor after different treatments indicated

Encouraged by the strong MW heating effect of chitosan/Fe_3_O_4_@IL microcapsules presented in vitro experiment, we then investigated in vivo cancer MW therapy using chitosan/Fe_3_O_4_@IL microcapsules in a H22 tumor mouse model. Female Institute for Cancer Research (ICR) mice bearing H22 tumors were intratumorally injected with chitosan/Fe_3_O_4_@IL microcapsules (200 mg/kg) and then exposed to the MW irradiation at the power density of 1.8 W/cm^2^ for 5 min. Mice without treatment were used as the control group. The temperature changes on tumor were real-time monitored by an infrared thermal camera and a fiber thermometer during MW irradiation. It was uncovered that the temperature of the tumor injected with chitosan/Fe_3_O_4_@IL microcapsules rapidly increased to about 51.2 °C (ΔT = 25.0 °C) within 5 min under MW irradiation (Fig. 3a, b), which is enough to kill cancer cells [40, 41]. In marked contrast, tumors showed much less temperature change (41.3 and 40.9 °C, respectively).

Next, the in vivo therapeutic efficacy of microcapsules-induced MW cancer treatment was investigated. Four groups of H22 tumor-bearing mice with five mice per group were used in our experiment. For the treatment groups, tumors were intratumorally injected with chitosan/Fe_3_O_4_@IL microcapsules (200 mg/kg) and then irradiated by the MW at a power density of 1.8 W/cm^2^ for 5 min (Chitosan/Fe_3_O_4_@IL + MW). Other control groups of mice included untreated mice (Control), mice with chitosan/Fe_3_O_4_@ILmicrocapsule injection without MW irradiation (Chitosan/Fe_3_O_4_@IL), and mice with MW irradiation without chitosan/Fe_3_O_4_@IL microcapsules injection (MW). The tumor sizes were measured by a caliper every 3 days after treatment. As shown in Fig. 3c, d and Additional file 1: Figure S4, we found that tumors treated with chitosan/Fe_3_O_4_@ILmicrocapsules were effectively ablated after MW irradiation. However, tumor in other control groups showed partially damaged and exhibited a similar growth speed, suggesting that either MW irradiation (1.8 W/cm^2^) or chitosan/Fe_3_O_4_@IL microcapsule injection by itself does not affect the tumor development (Fig. 3c, d and Additional file 1: Figure S4). Our results demonstrate that chitosan/Fe_3_O_4_@IL microcapsules would be a powerful MW agent for effective tumor ablation.

### MR Imaging

**Fig. 4:**
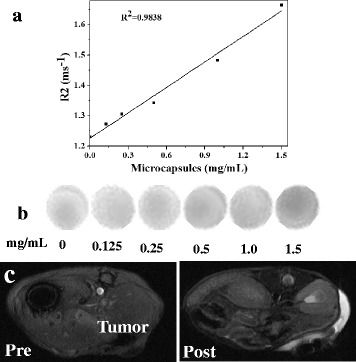
Magnetic properties of chitosan/Fe_3_O_4_@ILmicrocapsules. **a** The T_2_ relaxation rates (*r*_2_) of chitosan/Fe_3_O_4_@IL microcapsules at different concentrations. **b** T_2_-weighted MR images of chitosan/Fe_3_O_4_@IL. **c** T_2_-weight MR imaging of H22 tumor-bearing mice before (*left*) and after (*right*) intratumoral injection of chitosan/Fe_3_O_4_@IL microcapsules

MR imaging can provide imaging with good anatomical details and functional information with real-time monitoring manner. In consideration of that, Fe_3_O_4_-based particles have the potential to be used as contrast agent for T_2_ MR imaging. To explore the possibility of using chitosan/Fe_3_O_4_@IL microcapsules as a T_2_-weighted MR contrast agent, we measured its magnetic properties and T_2_ relaxation time. The magnetic curves of the chitosan/Fe_3_O_4_@IL microcapsules obtained at room temperature (Additional file 1: Figure S5) showed the saturation magnetization of 0.55 eumg^−1^. When placed a magnet, the chitosan/Fe_3_O_4_@IL microcapsules was rapidly attracted by the magnet (inset of Additional file 1: Figure S5). As presented in Fig. 4a b, an obvious concentration-dependent darkening effect in T_2_-weighted MR images acquired by a 3.0 T magnetic resonance system at room temperature was observed for chitosan/Fe_3_O_4_@IL microcapsules. The T_2_ relaxivity (r_2_) of chitosan/Fe_3_O_4_@IL microcapsules was measured to be 0.27943 mg^−1^ mL s^−1^. Next in vivo experiments were used to characterize chitosan/Fe_3_O_4_@IL microcapsules as a MR imaging probe. Female ICR mice bearing H22 tumors were intratumorally injected with chitosan/Fe_3_O_4_@IL microcapsules (200 mg/kg) and imaged by a 3.0 T MR imaging instrument. As presented in Fig. 4c, an obviously darkened T_2_ MR signals decrease of 32.27 % in the tumor appeared, indicating that our microcapsules would be a promising candidate as a contrast agent in MR imaging for cancers therapy.

### In Vivo Systematic Toxicity

**Fig. 5:**
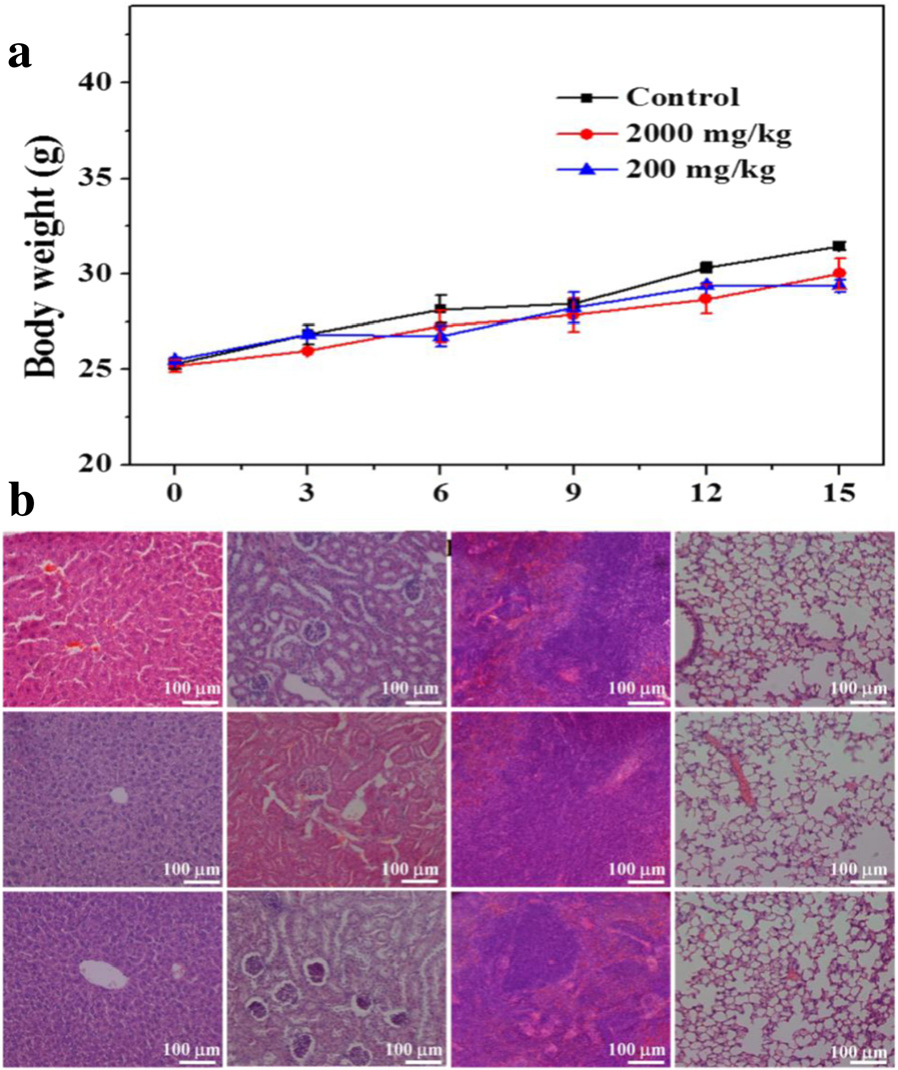
In vivo systematic toxicity. **a** Body weight curves from healthy ICR mice injected subcutaneously in the axillary region with 2000 mg/kg, 200 mg/kg, and without treatment. **b** H&E stained images of major organs. Healthy ICR mice injected with microcapsules were sacrificed 16 days. No noticeable abnormality was observed in major organs including the liver, kidney, spleen, and lung

To further study the chitosan/Fe_3_O_4_@IL microcapsules on the toxicity of target organs, healthy ICR mice were randomly divided into three groups (*n* = 5) and injected subcutaneously in the axillary region with 2000 mg/kg, 200 mg/kg, and without treatment. Then, all mice were sacrificed and collected their heart, liver, spleen, lung, and kidney organs for histological examination with standard techniques at day 16. Neither death nor significant body weight variation was noticed after microcapsules treatment during the treatment (Fig. 5a). There was no noticeable organ damage or inflammation in these groups (Fig. 5b). Although the systematic long-term toxicology of chitosan/Fe_3_O_4_@IL microcapsules in animals remain to be carefully examined, our preliminary results indicate that chitosan/Fe_3_O_4_@IL microcapsules may not be obviously toxic to mice.

## Conclusions

In summary, we have successfully fabricated a new generation of MW theranostic agent based on chitosan microcapsules, which could be used for MR imaging-guided MW thermotherapy of cancer. The hemolysis rate and HepG2 cells viability test with chitosan/Fe_3_O_4_@IL presented good biocompatibility when their concentration were less than 2000 mg/mL and less than 1000 mg/mL, respectively. The tumor cells were effectively ablated using low power density (1.8 W/cm^2^) upon a short irradiation time (5 min). Besides, the use of these well-prepared chitosan/Fe_3_O_4_@IL microcapsules as MR contrast agents was proved. Our design and the construction of chitosan/Fe_3_O_4_@IL microcapsules could provide more opportunities for further MW thermotherapy applications.

## Additional File

Additional file 1: Figures S1–S5.
**Figure S1**. Size distribution of chitosan, chitosan/Fe3O4 and chitosan/Fe3O4@IL microcapsules were determined by a panel of more than 200 objects in Figure 1b. **Figure S2**. EDS spectrum of chitosan, chitosan/Fe3O4 and chitosan/Fe3O4@IL microcapsules. **Figure S3**. TG curve of chitosan, chitosan/Fe3O4 and chitosan/Fe3O4@IL microcapsules. **Figure S4**. Tumor weight in different groups of mice after various treatments indicated. **Figure S5**. Magnetization loops of chitosan/Fe3O4@IL microcapsules. **Figure S6**. FT-IR spectra of IL. (DOC 259 kb)
