# Supplementary material for: In Vivo Magnetic Resonance Imaging and Microwave Thermotherapy of Cancer Using Novel Chitosan Microcapsules
Source: Nanoscale Res Lett. 2016 Jul 15;11:334. doi: 10.1186/s11671-016-1536-0 (PMC4947076; doi:10.1186/s11671-016-1536-0)
Supplement: Additional file 1: Figures S1–S5. — Figure S1. Size distribution of chitosan, chitosan/Fe3O4 and chitosan/Fe3O4@IL microcapsules were determined by a panel of more than 200 objects in Figure 1b. Figure S2. EDS spectrum of chitosan, chitosan/Fe3O4 and chitosan/Fe3O4@IL microcapsules. Figure S3. TG curve of chitosan, chitosan/Fe3O4 and chitosan/Fe3O4@IL microcapsules. Figure S4. Tumor weight in different groups of mice after various treatments indicated. Figure S5. Magnetization loops of chitosan/Fe3O4@IL microcapsules. Figure S6. FT-IR spectra of IL. (DOC 259 kb) [file 11671_2016_1536_MOESM1_ESM.doc]

**Additional file 1**

***In vivo* magnetic resonance imaging and microwave thermotherapy of cancer using novel chitosan microcapsules**

Shunsong Tang^1,2^†, Qijun Du^2^†, Tianlong Liu^2^, Longfei Tan^2^, Meng Niu^1^，Long Gao^1^, Zhongbing Huang^3^*, Changhui Fu^2^, Tengchuang Ma^1^, Xianwei Meng^2^* and Haibo Shao^1^*

1 Department of Radiology First Hospital of China Medical University, No.155 Nanjing North Road, Shenyang, 110001, P.R. China.

2 Laboratory of Controllable Preparation and Application of Nanomaterials, Center for Micro/nanomaterials and Technology & Key Laboratory of Photochemical Conversion and Optoelectronic Materials, Technical Institute of Physics and Chemistry, Chinese Academy of Sciences, Beijing, 100190, China.

3 College of Materials Science and Engineering, Sichuan University, Chengdu, 610065, China.

* Corresponding author

Email: [haiboshao@aliyun.com](mailto:haiboshao@aliyun.com), [mengxw@mail.ipc.ac.cn](mailto:mengxw@mail.ipc.ac.cn)

†These authors contributed equally to this work.


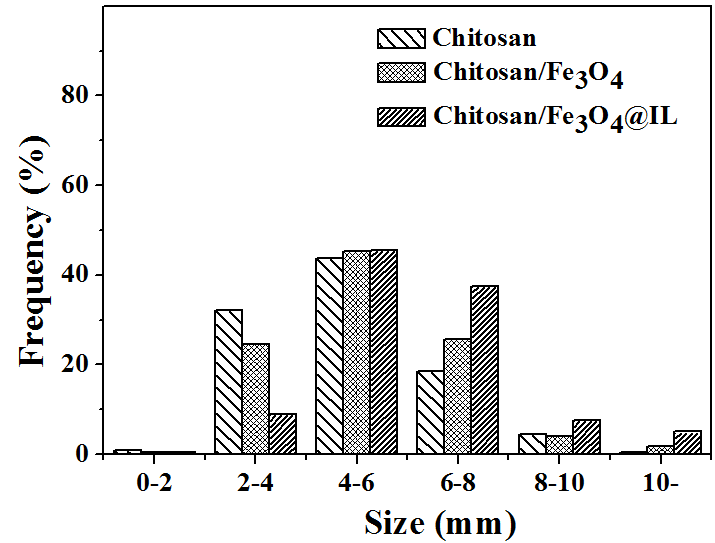


**Figure S1.** Size distribution of chitosan, chitosan/Fe_3_O_4_ and chitosan/Fe_3_O_4_@IL microcapsules were determined by a panel of more than 200 objects in Figure 1b.


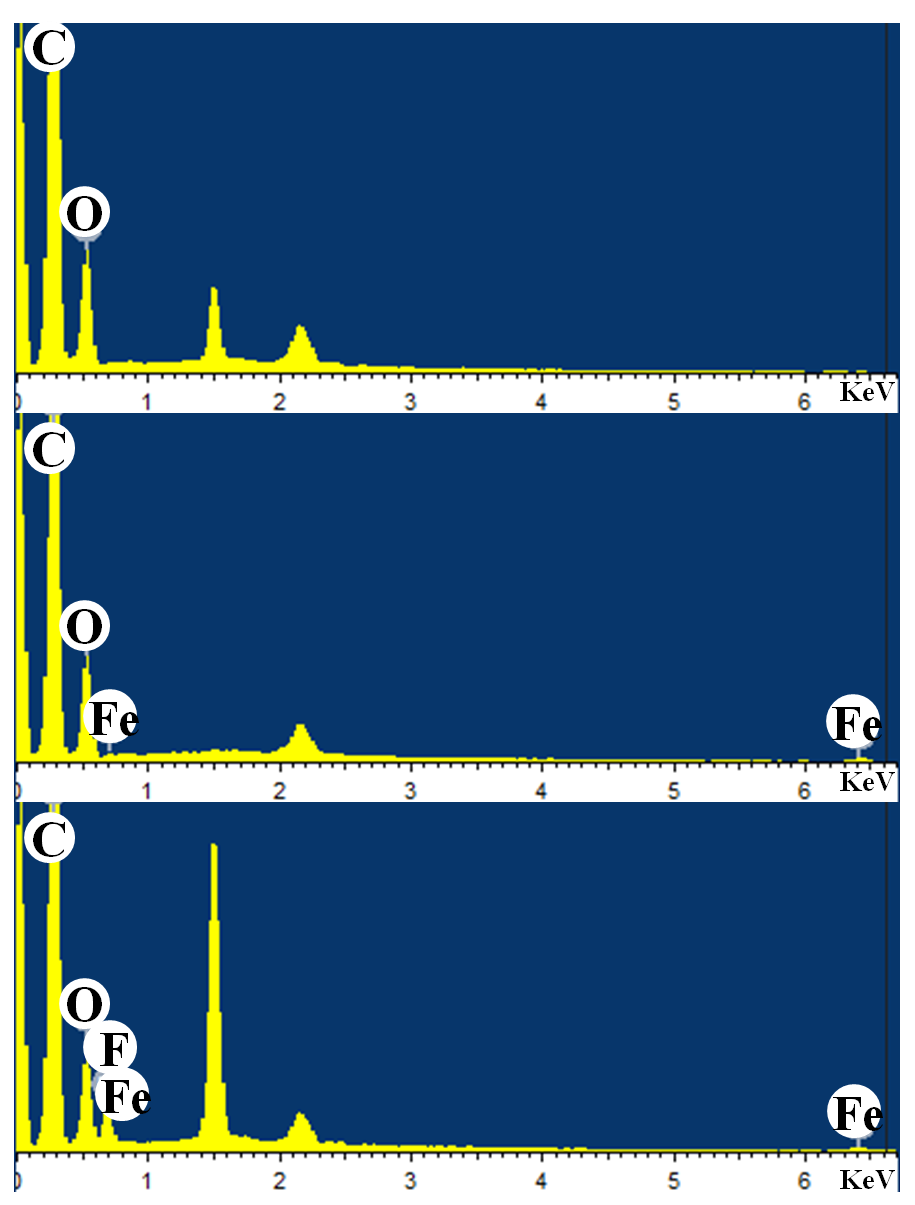


**Figure S2.** EDS spectrum of chitosan, chitosan/Fe_3_O_4_ and chitosan/Fe_3_O_4_@IL microcapsules.


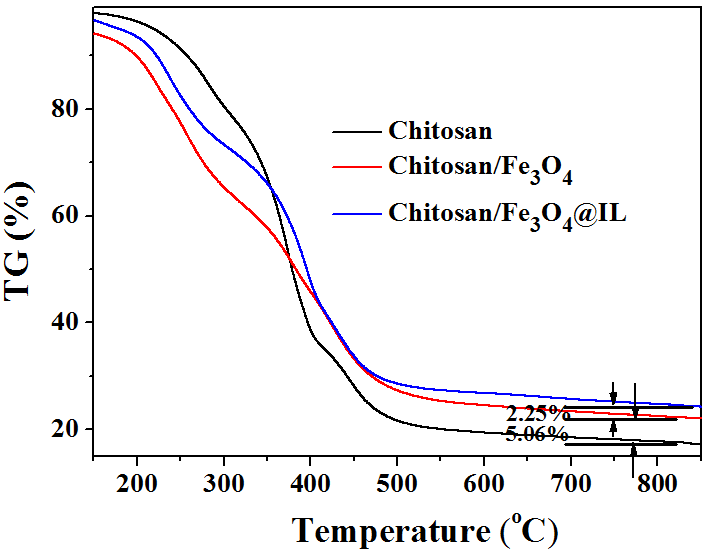


**Figure S3.** TG curve of chitosan, chitosan/Fe_3_O_4_ and chitosan/Fe_3_O_4_@IL microcapsules.


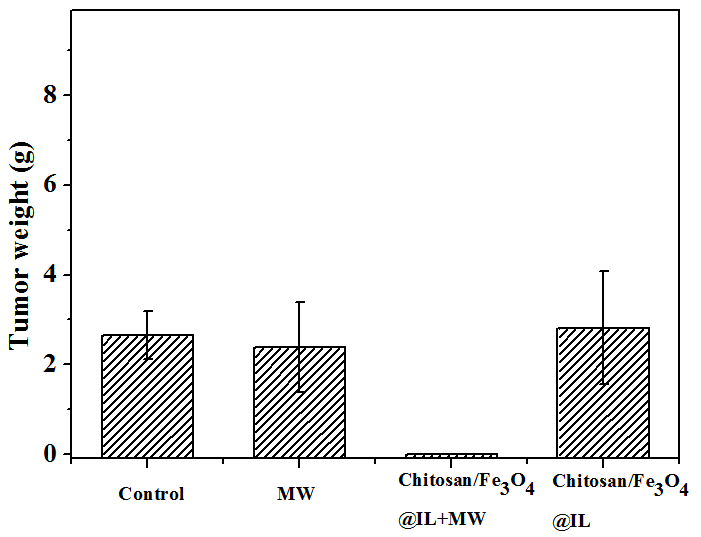


**Figure S4.** Tumor weight in different groups of mice after various treatments indicated.


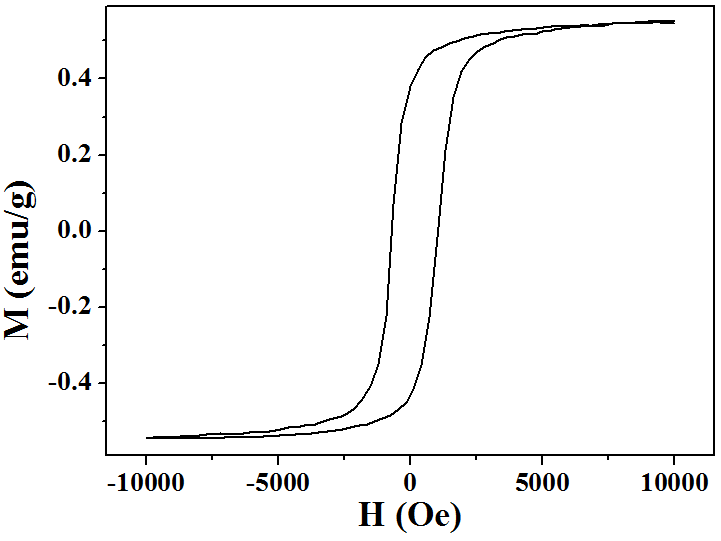


**Figure S5.** Magnetization loops of chitosan/Fe_3_O_4_@IL microcapsules.


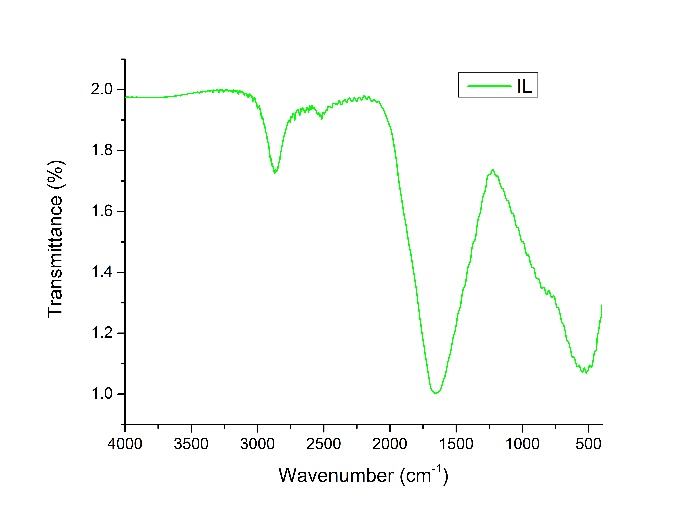


**Figure S6.** FT-IR spectra of IL.
